# Development and validation of a novel risk score to predict overall survival following surgical clearance of bilobar colorectal liver metastases

**DOI:** 10.1093/bjsopen/zrad085

**Published:** 2023-09-21

**Authors:** Bobby V M Dasari, Dimitri Raptis, Nicholas Syn, Alejandro Serrablo, Jose Manuel Ramia, Andrea Laurenzi, Christian Sturesson, Timothy M Pawlik, Ajith K Siriwardena, Mickael Lesurtel, BiCRLM study collaborators, Alexander Novotny, Alfred Kow, Amar Kourdouli, Andrea Belli, Andres Valdivieso, Angus Hann, Ángela de la Hoz Rodríguez, Anisa Nutu Oona, Andreas Pascher, Antonio Frena, Arpad Ivanecz, Asmus Heumann, Ayaya Alonso Alvarado, Ayrat Kaldarov, Bart Bracke, Bart Hendrikx, Benjamin Struecker, Bergthor Bjornsson, Carmen Cutolo, Carlo Frola, Carmen Payá-Llorente, Carlos Domingo-del Pozo, Catherine Teh, Christian Stöss, Claudio Ricci, Cornelis Verhoef, Cristina Dopazo, Daniel Galun, Daniel Hartmann, David Martin, Diego Greatti Vaz da Silva, Dimitri Dorcaratto, Dimitrios Magouliotis, Dimitrios Moris, Dimitrios Symeonidis, Dimitrios Zacharoulis, Dursun Bugra, Dolores Lopez-Garnica, Eduard Jonas, Edoardo Maria Muttillo, Edoardo Saladino, Elsa Francisco, Ela Hutten, Emilio De Raffele, Emanuele Felli, Emre Balik, Emre Bozkurt, Evangelos Felekouras, Erman Sobutay, Ernesto Sparrelid, Fabrizio Romano, Felipe José Fernández Coimbra, Fiorentini Guido, Florian Primavesi, Francesco Izzo, Frederik Berrevoet, Gaetano Piccolo, Gaëtan-Romain Joliat, Gary Middleton, Georgios Makridis, Georgios C Sotiropoulos, Giuseppe Garcea, Glen Booney, Ho-Seong Han, Ibrahim Halil Ozata, Jai Young Cho, Jiri Pudil, John Hammond, Jorge Brian Torres, Jun Li, Joerg-Matthias Pollok, Khaled Ammar, Kostiantun Kopchak, Kojiro Taura, Kursat Serin, Krishna Menon, Krzysztof Zieniewicz, Leticia Perez-Santiago, Linda Lundgren, Lissa Wullaert, Luca Alderghetti, Luis Abreu De Carvalho, Madita-Magdalena Tschöegl, Marco Marino, María Aránzazu, Markus Ammann, Aranzazu Varona-Bosque, Mario Giuffrida, Mattia Garancini, Mauro Alessandro Scotti, Matteo Barabino, Marc Bernon, Matteo Cescon, Marcello Di Martino, Marcello Maestri, Marco Massani, Maria Sotiropoulou, Maria Teresa Abadia Forcen, Maria-Carmen Fernandez-Moreno, Mario Serradilla-Martín, Marko Zivanovic, Marta Gutiérrez-Díez, Melek Buyuk, Michail Vailas, Mitesh Sharma, Mizelle D'Silva, Mladjan Protic, Mohammad Hossein Fard-Aghaie, Lissa Wullaert, Nagappan Kumar, Narimã Marques, Nefeli Tomara, Nicholas G Mowbray, Nicolas Demartines, Nikolaos Machairas, Offir Ben-Ishay, Oleksandr Kvasivka, Olivera Krsmanovic, Orhan Bilge, Pablo Sancho-Pardo, Pal-Dag Line, Pascale Tinguely, Patrick Pessaux, Per Sandstrom, Peter Lodge, Raffaele Dalla Valle, Roger Homs, Robert Sutcliffe, Sanja Lob, Santiago Sánchez-Cabús, Shadi Katou, Shinya Okumura, Etsuro Hatano, Spela Turk, Stefan Farkas, Stefan Patauner, Stefan Stättner, Stefan Löb, Stephanie Truant, Stylianos Kapiris, Tom Gallagher, Tereza Kocisova, Thomas Gruenberger, Tommaso Stecca, Thiery Chapelle, Teresa Abadía-Forcén, Víctor Molina, Valeriia Sumarokova, Yannick Meyer

**Affiliations:** Institute of Immunology and Immunotherapy, University of Birmingham, Birmingham, UK; Department of HPB Surgery and Liver Transplantation, Queen Elizabeth Hospital, Birmingham, UK; Department of HPB Surgery and Liver Transplantation, Royal Free Hospital, London, UK; Department of HPB Surgery and Liver Transplantation, National University of Singapore, Singapore; HBP Surgical Division, Miguel Servet University Hospital, Zaragoza, Spain; Department of Hepatobiliary Surgery and Liver Transplantation, Hospital General Universitario de Alicante, Alicante, Spain; Hepatobiliary Surgery and Organ Transplantation, IRCCS Azienda Ospedaliero-Universitaria di Bologna, Bologna, Italy; Division of Surgery, Department of Clinical Science, Intervention and Technology (CLINTEC), Karolinska Institutet and Karolinska University Hospital, Stockholm, Sweden; Division of Surgery, Oncology, and Health Services Management and Policy, The Ohio State University Wexner Medical Center, Ohio, USA; Department of Hepatobiliary Surgery, Manchester Royal Infirmary, Manchester, UK; Department of HPB Surgery & Liver Transplantation, Beaujon Hospital—University of Paris Cité, Paris, France

## Abstract

**Background:**

Bilobar liver metastases from colorectal cancer pose a challenge for obtaining a satisfactory oncological outcome with an adequate future liver remnant. This study aimed to assess the clinical and pathological determinants of overall survival and recurrence-free survival among patients undergoing surgical clearance of bilobar liver metastases from colorectal cancer.

**Methods:**

A retrospective international multicentre study of patients who underwent surgery for bilobar liver metastases from colorectal cancer between January 2012 and December 2018 was conducted. Overall survival and recurrence-free survival at 1, 2, 3 and 5 years after surgery were the primary outcomes evaluated. The secondary outcomes were duration of postoperative hospital stay, and 90-day major morbidity and mortality rates. A prognostic nomogram was developed using covariates selected from a Cox proportional hazards regression model, and internally validated using a 3:1 random partition into derivation and validation cohorts.

**Results:**

A total of 1236 patients were included from 70 centres. The majority (88 per cent) of the patients had synchronous liver metastases. Overall survival at 1, 2, 3 and 5 years was 86.4 per cent, 67.5 per cent, 52.6 per cent and 33.8 per cent, and the recurrence-free survival rates were 48.7 per cent, 26.6 per cent, 19.2 per cent and 10.5 per cent respectively. A total of 25 per cent of patients had recurrent disease within 6 months. Margin positivity and progressive disease at liver resection were poor prognostic factors, while adjuvant chemotherapy in margin-positive resections improved overall survival. The bilobar liver metastases from colorectal cancer-overall survival nomogram was developed from the derivation cohort based on pre- and postoperative factors. The nomogram’s ability to forecast overall survival at 1, 2, 3 and 5 years was subsequently validated on the validation cohort and showed high accuracy (overall C-index = 0.742).

**Conclusion:**

Despite the high recurrence rates, overall survival of patients undergoing surgical resection for bilobar liver metastases from colorectal cancer is encouraging. The novel bilobar liver metastases from colorectal cancer-overall survival nomogram helps in counselling and informed decision-making of patients planned for treatment of bilobar liver metastases from colorectal cancer.

## Teaser

At a median follow-up of 50.9 months, the 1-year, 2-year, 3-year and 5-year overall survival rates were 86.4 per cent, 67.5 per cent, 52.6 per cent and 33.8 per cent respectively; the corresponding recurrence-free survival rates were 48.7 per cent, 26.6 per cent, 19.2 per cent and 10.5 per cent; the study demonstrates survival advantage of adjuvant chemotherapy in patients with margin-positive resection.

## Introduction

Approximately 50 per cent of patients with colorectal cancer develop liver metastases during the course of the disease^1^, with only 20–25 per cent of patients with colorectal liver metastases (CRLM) deemed resectable at initial presentation^2–4^. Multimodality therapy including complete surgical resections remains the best treatment strategy^5^ and patients eligible for surgical resection can experience 5- and 10-year survival rates of approximately 50 per cent and 25 per cent respectively^6,7^.

CRLM involving both lobes of the liver, bilobar CRLM (BiCRLM), form a particularly challenging clinical situation. Compared with unilobar disease, patients with BiCRLM have a greater mean number of tumours, are more likely to have an advanced primary tumour stage at presentation, and be more prone to R1 resection. Consequently, these patients tend to have a worse overall survival (OS) and higher recurrence rates^8–11^. Resection of BiCRLM is challenging because it can be difficult to achieve margin-negative resection while preserving sufficient functional liver parenchyma to avoid postoperative hepatic insufficiency. It was also reported that patients with four or more CRLM are likely to have a particularly poor prognosis^12^.

Surgical management options for patients with BiCRLM are based on the size, location, and distribution of the lesions; proximity to the portal and hepatic vein branches; and preservation of an adequate future liver remnant. Surgeons worldwide have expanded the treatment of BiCRLM by innovative combinations of anatomic hepatectomy, wedge resections, one-stage parenchymal sparing hepatectomy, two-stage hepatectomy with or without portal vein embolization, double vein embolization and the associating liver partition and portal vein ligation for staged hepatectomy (ALPPS) procedures. Liver transplantation is being discussed as a potential option for the management of patients with CRLM who have favourable disease behaviour^13–15^. However, a mortality rate of 10 per cent and a severe morbidity rate of 25 per cent were reported for some of these procedures^16,17^. Further, there is an additional risk of early recurrence following clearance of BiCRLM^9,18^. With such high morbidity, mortality, and recurrence rates among patients with BiCRLM, there is a need for better risk stratification that will allow prediction of survival following surgical resection. None of the currently employed risk scores that are used to predict OS following resection of CRLM were developed exclusively in the context of bilobar disease^19–22^. Extensive BiCRLM also raises the possibility of an unfavourable tumour microenvironment. For these reasons, BiCRLM need to be treated as a different entity to standard colorectal cancer liver metastases with critical assessment of the benefit of surgical resection.

The aim of the current study was to identify the clinical and pathological determinants of OS and recurrence-free survival (RFS) in patients undergoing surgical treatment for BiCRLM and then develop a nomogram that can be used to predict OS following surgical clearance of BiCRLM.

## Methods

An international retrospective multicentre study supported by the European-African Hepato-Pancreato-Biliary Association (E-AHPBA) was performed including patients who underwent liver resection for BiCRLM between January 2012 and December 2018. Each registered centre appointed one dedicated contact person who registered details for the study. Predefined electronic case report forms (CRF) were used for data collection from all participating centres (*form S1*). The study protocol was registered on Research Registry (UIN: researchregistry8441). The study was approved by the E‐AHPBA Scientific and Research Committee.

### Inclusion criteria

Patients with at least two lesions on the anatomical right side and two lesions on the anatomical left side were included. Liver surgery needed to be performed with curative intent with planned clearance of liver disease by any combination of surgical and ablative procedures. Patients who had clearance of BiCRLM as well as individuals who failed to complete the surgical pathway (such as drop-outs after 1st stage procedures) were included in the study.

### Outcomes assessed

The primary outcomes were OS and recurrence-free survival (RFS) (measured at 1, 2, 3 and 5 years after surgery). The secondary outcomes were duration of postoperative hospital stay, and 90-day major morbidity and mortality rates. A prognostic nomogram was developed using covariates selected from a Cox proportional hazards regression model, and internally validated from a random partition of 3:1 into derivation and validation cohorts. The Transparent Reporting of a Multivariable Prediction Model for Individual Prognosis or Diagnosis (TRIPOD) statement was followed for development of the prediction model (*form S2*).

### Definitions used

R0 resection was defined as a tumour-free margin ≥1 mm from the metastatic lesion, R1 as a < 1 mm margin from the lesion, and R2 resection as a macroscopically positive margin of the liver metastases. For multiple resected lesions, the margin status of the lesion with the worst margin was recorded for analysis. Patients with multisite disease (such as lung metastases at liver resection) were documented as extrahepatic disease and not as R2. Synchronous disease was defined as the presence or development of liver metastases within 12 months of primary colorectal cancer diagnosis. AJCC cancer staging manual 7th or 8th edition of the AJCC Cancer Staging Manual was used for TNM staging of the primary tumour. Right hepatectomy included resection of segments 5, 6, 7, 8 and segments 1, 2, 3, 4 in left hepatectomy. The two-stage procedures included portal venous ligation (PVL), portal venous embolization (PVE) and/or dual vein [portal vein (PVE) and hepatic vein (HVE)] embolization (DVE) and associating liver partition and portal vein ligation for staged hepatectomy (ALPPS) procedures. Postoperative complications were classified using the Clavien–Dindo classification of surgical complications with major complications defined as Clavien–Dindo grade ≥ IIIa.

### Statistical analysis

Baseline and perioperative characteristics are summarized as median values (interquartile range) or fractions (percentages) for continuous and binary variables respectively. Overall survival was defined as the time from the resection of liver metastases to death and was censored at the last follow-up. Recurrence-free survival was defined as the time from surgery to the point of recurrence or death, whichever occurred first, and was censored at the last follow-up if no events occurred. Median follow-up was calculated using the reverse Kaplan–Meier method. The patients were randomly partitioned 3:1 into derivation and validation cohorts based on a uniform distribution. We selected prognostic covariates and manually generated interaction terms for inclusion in the penalized Cox proportional hazards regression model using an L1-regularized machine learning procedure based on the least absolute shrinkage and selection operator (LASSO) λ penalty, the optimal value of which was selected based on the minimum mean cross-validated error through 10-fold cross-validation in the derivation cohort. Area under receiver operating characteristic (AUROC) curve analyses were used to identify the binary cut-off value for the number of lesions influencing the OS. Coefficients from the LASSO–Cox model were imported into the ‘nomocox’ program to generate a nomogram to obtain personalized predictions of patients’ survival based on points scoring systems. Since a fraction of patients with colorectal liver metastases will experience a ‘cure’, which manifests as a long plateau at the tail ends of Kaplan–Meier curves, parametric cure models were also examined. In particular, the lognormal accelerated failure-time (AFT) model provided excellent goodness-of-fit and hazard ratios that were nearly identical to the semi-parametric multivariable Cox proportional hazards model. Estimates from the lognormal AFT model were therefore used to predict the expected ‘cured’ proportions and modelled OS curves. Calibration was predominantly assessed at clinically relevant time points (1, 2, 3 and 5 years), and discrimination was assessed using Harrell’s C-index as well as the area under the curve at specified time points using the nearest neighbour method. All analyses were performed using Stata (version 16.1, StataCorp, College Station, TX, USA). Missing data were excluded on a complete case basis.

## Results

A total of 1257 patients from 70 participating units from 13 countries in Europe, South Korea, Japan and Brazil fulfilled the inclusion criteria, of which 1236 were included in the analysis (*Table S1*). In total, 21 patients were excluded due to incompletely returned CRFs. Patient-related, oncological and surgical characteristics in the overall cohort as well as the derivation and validation cohorts are provided in *Table 1*. The median patient age was 61 years (range: 21–89) and most patients were male (*n* = 786; 63.6 per cent). Right-sided primary tumour localization was reported in 19.3 per cent (*n* = 239) of patients. The primary tumour stage was T3 and T4 disease in 67.9 per cent (*n* = 767) and 19.5 per cent (*n* = 220) of patients respectively. A significant subset of patients had lymph node metastasis with N1 disease in 45.0 per cent (*n* = 504) and N2 nodal status in 30.6 per cent (*n* = 343); 88.5 per cent of patients had synchronous CRLM (*n* = 1089) and the primary tumour was *in situ* at the time of liver resection in 36 per cent of patients (*n* = 445). KRAS mutations were encountered in 23.9.% (*n* = 295), BRAF in 3.4 per cent (*n* = 42), and PIK3CA in 0.4 per cent (*n* = 5) of primary tumours respectively. Mutational status was unknown in the primary tumour and liver metastases in 48.2 per cent (*n* = 596) and 73 per cent (*n* = 902) of the patients respectively, indicating a lack of universality in the assessment of RAS mutational status during the study interval. Sixty-one per cent of patients received chemotherapy following primary resection (*n* = 754), 78.0 per cent received chemotherapy before liver resection (*n* = 975), and 62.4 per cent received chemotherapy after liver resection (*n* = 694). The time to diagnosis of synchronous metastases was 86 per cent < 3 months, 6 per cent 3–6 months, and 8 per cent 6–12 months. Within these synchronous groups, chemotherapy was given before liver resection in 81 per cent, 87 per cent, and 71 per cent respectively (*Table 2*). Most patients underwent single-stage liver resections (78 per cent), predominantly multiple wedge resections (*n* = 532; 43 per cent), followed by extended right/left or right/left hepatectomies (*n* = 210; 19.7 per cent), and a combination of options by single-stage resection (*n* = 191; 15.5 per cent). The remaining 21.8 per cent (*n* = 269) of patients underwent two-stage resection procedures. R0 margin status was reported in 68.0 per cent of the liver resections (*n* = 841), R1 in 28.2 per cent (*n* = 349) and R2 in 3.7 per cent (*n* = 46).

**Table 1:** Baseline and perioperative characteristics

Characteristic	Overall cohort (*n* = 1236)	Derivation cohort (*n* = 927)	Validation cohort (*n* = 309)
Median age, years (i.q.r.)	61 (54–69)	61 (54–68)	62 (54–69)
Male gender (*n*/total, %)	786 (63.6%)	578 (62.4%)	208 (67.3%)
Median BMI, kg/m^2^ (i.q.r.)	25.4 (23.0–27.8)	25.4 (23.0–27.7)	25.5 (23.0–28.3)
**ASA status (*n*/total, %)**			
I/II	879 (71.1%)	655 (70.7%)	224 (72.5%)
III/IV	357 (28.9%)	272 (29.3%)	85 (27.5%)
**Site of primary (*n*/total, %)**			
Ascending colon	239 (19.3%)	172 (18.5%)	67 (21.7%)
Transverse colon	43 (3.5%)	33 (3.6%)	10 (3.2%)
Descending colon	544 (44.0%)	421 (45.4%)	123 (39.8%)
Rectal	410 (33.2%)	301 (32.5%)	109 (35.3%)
**T-stage (*n*/total, %)**			
Tis	13/1129 (1.2%)	6/843 (0.7%)	7/286 (2.5%)
T1	18/1129 (1.6%)	15/843 (10.1%)	3/286 (1.1%)
T2	111/1129 (9.8%)	85/843 (10.1%)	26;286 (9.1%)
T3	767/1129 (67.9%)	575/843 (68.2%)	192/286 (67.1%)
T4	220/1129 (19.5%)	162/843 (19.2%)	58/286 58.3%)
**N-stage (*n*/total, %)**			
N0	274/1121 (24.4%)	202/837 (24.2%)	72/284 (25.4%)
N1	504/1121 (45.0%)	375/837 (44.8%)	129/284 (45.4%)
N2	343/1121 (30.6%)	260/837 (31.1%)	83/284 (29.2%)
**KRAS, primary tumour (%)**			
Mutant	295 (23.9%)	226 (24.4%)	69 (22.3%)
Wild-type	345 (27.9%)	263 (28.4%)	82 (26.5%)
Unknown	596 (48.2%)	438 (47.2%)	158 (51.1%)
Adjuvant chemotherapy after primary resection (%)	754 (61.0%)	579 (62.5%)	175 (56.6%)
Unresected primary (*n*/total, %)	188 (15.2%)	140 (15.1%)	48 (15.5%)
**Status of the primary tumour at time of liver resection (*n*/total, %)**			
*In situ*	445 (36.0%)	337 (36.3%)	108/309 (35.5%)
Resected	778 (62.9%)	550 (62.6%)	298 (65.2)
Complete response to chemotherapy	13 (1.1%)	18 (1.1%)	3 (1.0%)
**Days from diagnosis of primary until diagnosis of liver metastases (*n*/total, %)**			
0–180 days	990/1230 (80.5%)	752/925 (81.3%)	238/305 (78.0%)
180–364 days	99/1230 (8.0%)	69/925 (7.5%)	30/305 (9.8%)
≥365 days (metachronous)	141/1230 (11.5%)	104/925 (11.2%)	37/305 (12.1%)
**Presence of lung metastases at time of diagnosis of liver metastases (*n*/total, %)**	90 (7.3%)	70 (7.6%)	20 (6.5%)
Median total number of metastases (i.q.r.)	7 (5–10)	7 (5–10)	7 (5–10)
Median number of metastases in right lobe (i.q.r.)	4 (2–6)	4 (2–6)	4 (2–6)
Median number of metastases in left lobe (i.q.r.)	3 (2–4)	3 (2–4)	3 (2–4)
Size of largest lesion (mm)	28 (19–45)	29 (19–45)	27 (20–45)
Chemotherapy before liver resection (%)	975 (78.9%)	737 (79.5%)	238 (77.0%)
**RECIST (if received neoadjuvant chemotherapy) (*n*/total, %)**			
Complete response	21/886 (2.4%)	17/673 (2.5%)	4/213 (1.9%)
Partial response	632/886 (71.3%)	476/673 (70.7%)	156/213 (73.2%)
Stable disease	166/886 (18.7%)	123/673 (18.3%)	43/213 (20.2%)
Progressive disease	67/886 (7.6%)	57/673 (8.5%)	10/213 (4.7%)
**Type of resection (%)**			
Two-stage with PVE+/−HVE	163 (13.2%)	117 (12.6%)	46 (14.9%)
Two-stage with PVL	29 (2.4%)	21 (2.3%)	8 (2.6%)
Two-stage with ALPPS	77 (6.2%)	64 (6.9%)	13 (14.2%)
Right	81 (6.6%)	63 (6.8%)	18 (5.8%)
Left	81 (6.6%)	66 (7.1%)	15 (14.9%)
Extended right	26 (1.8%)	18 (1.9%)	8 (2.6%)
Extended left	22 (1.8%)	15 (1.6%)	7 (2.3%)
Multiple wedges	532 (43.0%)	390 (42.1%)	142 (46.0%)
Anatomical + wedge	191 (15.5%)	147 (15.9%)	44 (14.2%)
Others	34 (2.8%)	26 (2.8%)	8 (2.6%)
**Margin status (%)**			
R0	841 (68.0%)	623 (67.2%)	218 (70.6%)
R1	349 (28.2%)	268 (28.9%)	81 (26.2%)
R2	46 (3.7%)	36 (3.9%)	10 (3.2%)
Intraoperative ablation (*n*/total, %)	442 (35.85%)	325 (35.1%)	117 (37.9%)
90-day major complications (*n*/total, %)	246 (19.9%)	193 (20.8%)	53 (17.2%)
Median postoperative duration of hospital stay, days (i.q.r.)	10 (7–16)	10 (7–16)	10 (7–16)
Adjuvant chemotherapy after liver resection (*n*/total, %)	694/1113 (62.4%)	516/829 (62.2%)	178/284 (62.7%)

i.q.r., interquartile range.

**Table 2:** Percentage of patients who received chemotherapy in relation to resection of primary and BiCRLM

	Percentage of patients who received chemotherapy			
Synchronous* (*n* = 1089)	Before primary resection	After primary resection	Before resection of liver metastases	After resection of liver metastases
<1 month (*n* = 891)	42%	59%	81.5%	56.7%
1–3 months (*n* = 46)	17%	83%	87%	74%
3–6 months *(n* = 53)	24.5%	68%	70%	58%
6–12 months (*n* = 99)	27%	49%	56%	47%
Metachronous (*n* = 141)	13%	74.5%	73%	55%

* Synchronicity based on the time from diagnosis of primary to diagnosis of liver metastases.

At a median follow-up of 50.9 months, the 1-year, 2-year, 3-year and 5-year OS rates were 86.4 per cent, 67.5 per cent, 52.6 per cent and 33.8 per cent respectively. The corresponding RFS rates were 48.7 per cent, 26.6 per cent, 19.2 per cent and 10.5 per cent respectively. Early recurrence rates at the 3-month and 6-month follow-up were 12.8 per cent and 28.0 per cent respectively. The treatment of patients with recurrent disease included repeat surgery in 234 patients (+chemotherapy in 115; +ablation in 63), ablation in 145 patients (+chemotherapy in 69), stereotactic ablative body radiotherapy in 18, selective internal radiotherapy in 14, and systemic chemotherapy in 537.

Right-sided lesions and N2 nodal status showed a negative influence on OS (HR 1.35 (1.07–1.71) and HR 1.31 (1.09–1.58) respectively). In addition, N1 and N2 nodal status negatively influenced RFS (HR 1.39 (1.19–1.61)). Patients with synchronous CRLM had a worse prognosis than those with metachronous liver disease (HR 0.71 (0.54–0.95)). The primary tumour was *in situ* at the time of liver resection in 36.0 per cent (*n* = 445) of patients with no influence on OS (HR 1.11 (0.90–1.36)) but had a negative influence on RFS (HR 1.42 (1.18–1.72)). Chemotherapy administered before liver resection had no influence on OS and RFS (HR 1.09 (0.90–1.32) and HR 1.12 (0.96–1.32) respectively). Disease progression while on chemotherapy was associated with a worse OS (HR 1.87 (1.28–2.47)) but not so for RFS (HR 1.22 (0.91–1.63)). The median size of the largest liver lesion was 2.8 cm (i.q.r. 1.9–4.5 cm). At least four lesions were present in 18 per cent, 5–10 lesions in 58 per cent, and more than 10 lesions in the remaining 24 per cent. Based on the AUROC analysis of the distribution of lesions, outcome analysis was performed in patients with <5 and ≥5 lesions (AUROC 0.65). More than five lesions proved to be an adverse factor for OS (HR 1.30 (1.07–1.57)) and RFS (HR 1.55 (1.35–1.78)). Two-stage resections had a worse OS in this cohort compared with single-stage resections (HR 1.51(1.23–1.85)). Grade IIIa or higher postoperative complications were encountered in 20 per cent of patients and were associated with worse OS (HR 1.52 (1.31–2.66)). Overall 90-day mortality rate in the cohort was 1.9 per cent. R1 and R2 margin status was associated with worse OS (HR 1.67 (1.27–2.20) and 12.59 (5.67–27.95) respectively) and RFS (HR 1.95 (1.51–2.52) and 2.83 (1.04–7.68) respectively). Fifty-four per cent of R1 and 58 per cent of R2 patients received adjuvant chemotherapy following liver resection. Adjuvant chemotherapy in patients with R1 resection had a protective effect on OS (HR 0.67 (0.46–0.97)) and RFS (HR 0.61 (0.44–0.84)). Similarly, in patients with adjuvant chemotherapy after R2 resection HRs for the OS were 0.18 (0.07–0.49) and RFS 0.64 (0.21–2.00) respectively. The unadjusted as well as adjusted HRs for all tested prognostic factors for OS and RFS from the final multivariate Cox models are shown in *Table 3*.

**Table 3:** Adjusted and unadjusted HRs of candidate variables for multivariable survival models

	Overall survival		Recurrence-free survival	
Covariate	Unadjusted HR (95% c.i.)	Adjusted HR (95% c.i.)	Unadjusted HR (95% c.i.)	Adjusted HR (95% c.i.)
Age ≥ 65 *versus* < 65 years	1.45 (1.25–1.70)	1.45 (1.21–1.73)	1.16 (1.02–1.33)	1.18 (1.02–1.36)
Male *versus* female gender	1.20 (1.02–1.41)		1.13 (0.99–1.30)	
BMI (per 1.0 kg/m^2^)	0.99 (0.97–1.01)		0.99 (0.98–1.02)	
ASA III–IV *versus* I–II	1.11 (0.94–1.32)	1.23 (1.01–1.48)	1.05 (0.91–1.21)	
**Site of primary tumour (*n*/total, %)**				
Left	Ref	Ref	Ref	
Rectum	1.09 (0.92–1.30)	1.06 (0.87–1.29)	1.04 (0.90–1.21)	
Right	1.25 (1.01–1.54)	1.35 (1.07–1.71)	1.04 (0.87–1.24)	
Transverse	1.60 (1.09–2.36)	1.61 (1.05–2.47)	1.04 (0.73–1.48)	
**T-stage**				
Tis	Ref		Ref	
T1	1.15 (0.40–3.32)		1.58 (0.70–3.57)	
T2	1.14 (0.46–2.86)		1.68 (0.84–3.33)	
T3	1.30 (0.54–3.14)		1.38 (0.71–2.67)	
T4	1.71 (0.70–4.19)		1.82 (0.93–3.54)	
N2 *versus* (N0 & N1)	1.56 (1.31–1.85)	1.31 (1.09–1.58)	1.47 (1.28–1.70)	1.39 (1.19–1.61)
Adjuvant chemotherapy after primary resection	0.82 (0.71–0.96)		1.00 (0.88–1.15)	
Unresected primary	1.11 (0.90–1.36)		1.67 (1.42–1.98)	1.42 (1.18–1.72)
**Status of the primary tumour at the time of liver resection**				
*In situ*	Ref		Ref	
Resected	0.89 (0.76–1.04)		0.88 (0.77–1.00)	
Complete response to chemotherapy	0.79 (0.33–1.92)		0.92 (0.47–1.78)	
Metachronicity *versus* synchronicity	0.67 (0.52–0.82)	0.71 (0.54–0.95)	0.75 (0.62–0.93)	
Presence *versus* absence of lung metastases at the time of diagnosis of liver metastases	0.99 (0.75–1.32)		1.06 (0.82–1.36)	
Total number of liver metastases (> 5 *versus* ≤ 5)	1.46 (1.23–1.73)	1.30 (1.07–1.57)	1.55 (1.35–1.78)	
Size of largest lesion (per 10 mm)	1.00 (0.98–1.02)		1.01 (1.00–1.03)	
Chemotherapy before liver resection	1.09 (0.90–1.32)		1.12 (0.96–1.32)	
RECIST (if received neoadjuvant chemotherapy) PD *versus* CR/PR/SD	1.78 (1.28–2.47)	1.87 (1.31–2.66)	1.22 (0.91–1.63)	
Two-stage *versus* one-stage resection	1.51 (1.26–1.80)	1.51 (1.23–1.85)	1.21 (1.03–1.41)	
**Margin status**				
R0	Ref	Ref	Ref	Ref
R1	1.45 (1.23–1.71)	1.67 (1.27–2.20)	1.50 (1.30–1.73)	1.95 (1.51–2.52)
R2	3.00 (2.06–4.38)	12.59 (5.67–27.95)	2.05 (1.36–3.08)	2.83 (1.04–7.68)
**Margin status and adjuvant chemotherapy after liver resection**				
R0 (if chemotherapy = yes)*	Ref	Ref	Ref	Ref
R1 (if chemotherapy = yes)*	1.01 (0.81–1.24)	0.67 (0.46–0.97)	1.25 (1.05–1.48)	0.61 (0.44–0.84)
R2 (if chemotherapy = yes)*	2.10 (1.29–3.40)	0.18 (0.07–0.49)	1.85 (1.14–2.99)	0.64 (0.21–2.00)
Intraoperative ablation	1.17 (1.01–1.37)		1.15 (1.01–1.32)	
Major complications	1.66 (1.39–1.20)	1.52 (1.31–2.66)	1.28 (1.09–1.51)	1.17 (0.98–1.40)
Adjuvant chemotherapy after liver resection	0.64 (0.54–0.75)	0.77 (0.62–0.96)	0.90 (0.78–1.04)	0.97 (0.81–1.16)

CR, complete response; PR, partial response; SD, stable disease; PD, progressive disease. *Unadjusted/univariable HRs for the interaction terms alone (that is not the full-factorial interaction) should not be interpreted by themselves and may also differ considerably from the HRs obtained from the full-factorial interaction in the multivariable analyses.

### Development and validation of the BiCRLM-OS nomogram

**Fig. 1:**
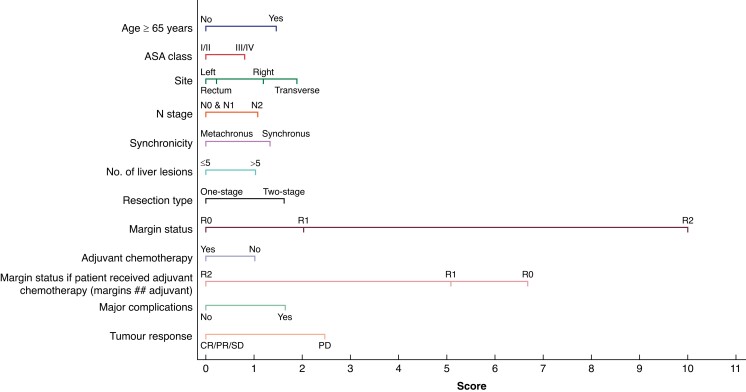
**BiCRLM-OS nomogram for the prediction of overall survival.** BiCRLM-OS, bilobar colorectal liver metastases-overall survival; CR, complete response; PR, partial response; SD, stable disease; PD, progressive disease.

A BiCRLM-OS nomogram was derived from the development cohort (*n* = 927) based on factors that significantly predicted OS in multivariate Cox regression analysis (*Fig. 1*). These factors included preoperative variables such as age, ASA grade, primary tumour factors (site, nodal status, synchronicity), metastasis-related factors (tumour load, margin status, chemotherapy, and response to chemotherapy), and presence of postoperative complications (*Table 4*).

**Table 4:** Factors included in the BiCRLM-OS nomogram and the (exponentiated) coefficients for the interaction terms

	Multivariable HR (95% c.i.)	Points
**Age**		
<65 years	Ref	+0.0
>/= 65 years	1.45 (1.21–1.73)	+1.5
**ASA**		
I & II	Ref	+0.0
III & IV	1.23 (1.01–1.48)	+0.8
**Site**		
Left	Ref	+0.0
Rectum	1.06 (0.87–1.29)	+0.2
Right	1.35 (1.07–1.71)	+1.2
Transverse	1.61 (1.05–2.47)	+1.9
**N status**		
N0 & N1	Ref	+0.0
N2	1.31 (1.09–1.58)	+1.1
**Timing**		
Synchronous	Ref	+1.3
Metachronous	0.71 (0.54–0.95)	+0.0
**Total number of liver lesions**		
≤ 5	Ref	+0.0
>5	1.30 (1.07–1.57)	+1.0
**Type of resection**		
One-stage	Ref	+0.0
Two-stage	1.51 (1.23–1.85)	+1.6
**Margins**		
R0	Ref	+0.0
R1	1.67 (1.27–2.20)	+2.0
R2	12.59 (5.67–27.95)	+10.0
**Adjuvant chemotherapy**		
No	Ref	+1.0
Yes	0.77 (0.62–0.96)	+0.0
**Protective effect of chemotherapy in margin-positive patients**		
R0	Ref	+6.7
R1	0.67 (0.46–0.97)	+5.1
R2	0.18 (0.07–0.49)	+0.0
**Major postoperative complications**		
No	Ref	+0.0
Yes	1.52 (1.31–2.66)	+1.6
**RECIST (if received neoadjuvant chemotherapy)**		
CR/PR/SD	Ref	+0.0
PD	1.87 (1.31–2.66)	+2.5

CR, complete response; PR, partial response; SD, stable disease; PD, progressive disease; Ref, reference.

**Fig. 2:**
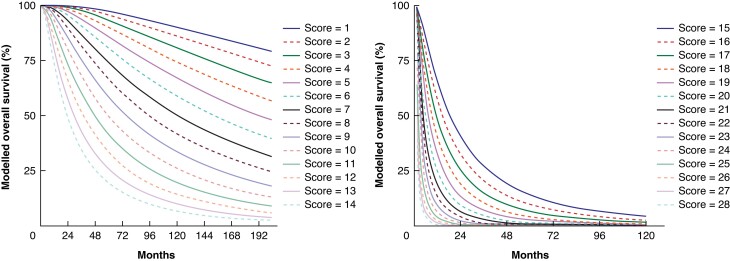
**Overall survival (OS) in the derivation cohort based on individual risk scores of BiCRLM-OS nomogram.** BiCRLM-OS, bilobar colorectal liver metastases-overall survival.

**Fig. 3:**
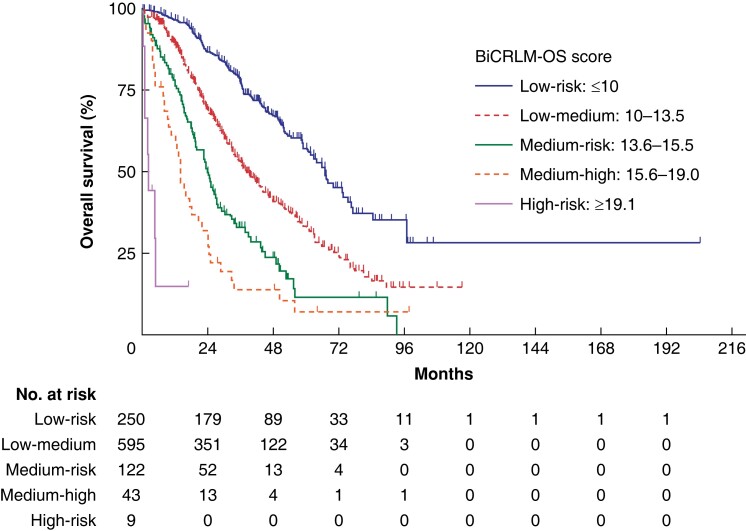
**Kaplan-Meier curves of the risk groups based on BiCRLM-OS risk score in the derivation cohort.** BiCRLM-OS, bilobar colorectal liver metastases-overall survival.

Based on the nomogram and the derived BiCRLM-OS score, patients were stratified into five risk groups: the median OS and predicted survival in the low-risk group (BiCRLM-OS score of ≤10), low-medium-risk group (score: 10–13.5), medium-risk group (score: 13.6–15.5), medium-high-risk group (score: 15.6–19.0), high-risk group (score of >19) and are shown in *Table S2*; *Fig. 2*. The cut-off values were based on the distribution of patients surviving for 5 years in the low-, medium- and high-risk groups and further stratification within the medium-risk group was performed based on the survival probability at 2 years. This was aimed at identifying the patients with good prognosis followed by those with a considerably worse prognosis. Kaplan-Meier curves based on the risk score are shown in *Fig. 3*. For example, a 70-year-old with synchronous BiCRLM from the right colonic primary tumour, who underwent a single-stage R1 resection for seven liver lesions, has a BiCRLM-OS score of 7. The predicted 5-year OS for a score of 7 is 73.5 per cent.

**Fig. 4:**
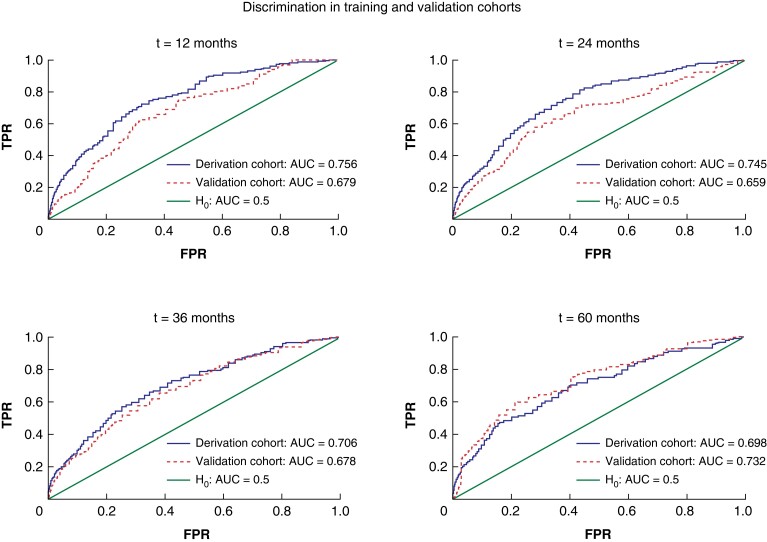
**Discrimination of BiCRLM-OS risk score for OS at 1-, 2-, 3- and 5-year intervals in the derivation and validation cohorts.** BiCRLM-OS, bilobar colorectal liver metastases-overall survival; AUC, area under the curve; TPR, true positivity rate; FPR, false positivity rate.

The OS nomogram exhibited clinically useful discrimination (overall C-index, 0.742). Time-dependent AUCs for 1-, 2-, 3- and 5-year follow-ups and calibration in the derivation and validation cohorts are shown in *Fig. 4*. No predictive nomogram for RFS reached a C-index with a useful discrimination of at least 0.70, therefore no such nomogram is reported. The online risk score can be accessed at: https://www.cognitoforms.com/BobbyDasari/ANovelRiskScoreToPredictOverallSurvivalFollowingSurgicalClearanceOfBilobarColorectalLiverMetastases.

## Discussion

The present study reports data from an international multicentre cohort to assess the clinical and pathological factors influencing OS after liver surgery for BiCRLM. The cohort specifically included only patients with at least two CRLM on each anatomical side of the liver that would typically require clearance on both sides of the liver and often challenge liver surgeons with respect to achieving clear margins with an adequate future liver remnant. A nomogram was developed to predict BiCRLM-OS in the derivation cohort based on patient, primary and secondary tumour characteristics and operative options, and validated showing high accuracy in the validation cohort. The BiCRLM-OS nomogram may help to forecast survival at 1, 3 and 5 years. This is particularly important, as the study demonstrated that 25 per cent of the patients had recurrent disease within 6 months and 72 per cent had recurrence within 2 years after liver resection and the OS in this cohort is much lower than the 30 per cent OS from previous published series^23^. In addition, a recent study reported the presence of underlying radiologically invisible occult disease in the explanted livers following liver transplantation colorectal liver metastases, indicating that the extent of liver disease in remnant liver is often underestimated^24^. Despite this, there is continued utilization of the different resection types by liver surgeons trying to push the boundaries for presumed curative liver resection. An estimate of OS is therefore important and none of the existing risk scores used in CRLM are developed exclusively from a cohort of patients with BiCRLM^19–22^. Some of the more recent scoring systems include gene profile status^25,26^ but this is not routinely performed as demonstrated in the current study. The current nomogram was exclusively developed from a cohort of patients with BiCRLM.

The surgical option to achieve complete clearance of BiCRLM can be challenging. While superficial lesions can be managed by multiple non-anatomical resections, deeper lesions, especially those in proximity to the vascular/biliary pedicles, require careful surgical planning^27–29^. In the current series, 45 per cent of the patients underwent multiple wedge resections. Findings from the present study demonstrated a worse OS in those who suffered major complications. Previous studies suggested that postoperative complications were associated with a worse outcome on median OS (74 *versus* 28 months, *P* <0.001) and median RFS (69 *versus* 23 months, *P* <0.001), possibly due to suppressed systemic immunity enabling disease dissemination^30^. A delay or lack of adjuvant chemotherapy administration is also a possible cause.

In a clinical setting in which wedge resections are not possible, a combination of anatomical/non-anatomical resections, sometimes combined with intraoperative ablation, is an option. A two-stage approach is considered when safe surgery is not feasible with a single-stage procedure. Two-stage resection is associated with a higher morbidity rate and failure to progress to complete resection^16,31^. In the current study, 20 per cent of patients underwent two-stage resection. Patients who underwent single-stage resection had better OS than those who underwent two-stage resection. While the exact reasons for the difference in survival could not be evaluated in the present study, higher tumour load in patients requiring two-stage resections or tumour progression between the stages of resection could be potential reasons.

A positive surgical margin at the time of liver resection remains a debatable issue, with R0 resections often achieved in only approximately 70–80 per cent of cases^11^. Higher margin positivity is also argued to be a factor resulting in worse outcomes with non-anatomical resection^32,33^. However, studies have shown that closer margins (up to 0.1 mm)^34,35^ and R1 status on vascular margins^36^ are associated with acceptable outcomes. RAS mutational status^23^ has also been noted to be more important in influencing OS than margin negativity as an independent factor, indicating the importance of achieving R0 resection in patients with RAS wild-type status. While all these factors are important, achieving a negative margin status should be considered the standard of care while deciding the surgical options of individual patients. The current study showed that R1 was associated with reduced OS, but R2 had a detrimental effect on outcomes, and R2 resection should not be accepted as a part of surgical planning for BiCRLM.

The role of perioperative chemotherapy in resectable CRLM remains debatable. A potential disadvantage of perioperative chemotherapy for CRLM is liver injury with the commonly used regimen^37^. However, in multivariate analysis, perioperative chemotherapy was an independent predictor of increased OS. Studies that evaluated the tumour burden in CRLM noted that perioperative chemotherapy increased OS in patients with a low risk of recurrence (*P* = 0.022)^38,39^. The role of chemotherapy in unresectable or borderline resectable CRLM is also well established with the aim of decreasing the tumour burden and achieving safe liver resection^40^. In the present study, progressive disease while on preoperative chemotherapy based on response evaluation criteria in solid tumours (RECIST) criteria had a deleterious effect on OS and RFS. Administration of adjuvant chemotherapy has shown a protective effect on the OS and RFS of patients with R2 and R1 resections.

Another risk factor influencing BiCRLM-OS was the presence of right-sided primary tumours and synchronous liver metastases. Right-sided colon cancers are more often diploid and hypermutated, frequently present with microsatellite instability, and have deleterious mutations in RAS, BRAF, and PI3KCa. This significant association between right-sidedness and worse OS after resection has been reported in the literature^41^. The present study also showed that having the primary *in situ* resected before the time of liver resection is associated with a better OS. It also concurs with previous studies^42^ on the negative impact of postoperative complications on OS after surgery for CRLM.

Recurrence rates at 2 years were higher in the present multicentre study (75 per cent) compared with rates of 40–60 per cent in the literature^42^. Calculation of a nomogram predicting RFS to identify patients at higher risk of recurrence was not possible, as no such model produced an adequate c-statistic. Factors associated with higher recurrence rates include higher T staging of primary, N2 status of primary, higher metastatic load, presence of primary *in situ* at liver resection, and R1/R2 status at liver resection. These factors must be considered in surgical planning, as previous studies have shown that early recurrence negatively affects prognosis: 5-year survival is 25.9 per cent for early recurrence *versus* 53.1 per cent for late recurrence (*P* <0.0001)^43,44^. Early recurrence can be attributed to the tumour microenvironment with micrometastases and circulating tumour cells, while missed lesions during surgery are also a possibility. Radical surgical options, such as liver transplantation, are currently evolving and may address the issue of liver replacement in carefully selected patients.

One of the limitations of this study was its retrospective design. Another limitation is that the current BiCRLM-OS lacks data regarding RAS mutations. RAS mutation has been linked to a more aggressive tumour pattern with decreased survival after hepatectomy and remains the basis of tumour biology evaluation. However, this information has not been routinely evaluated at many centres until recently, and the area of tumour biology continues to evolve rapidly with the identification of newer markers. The timing of chemotherapy in relation to resection of primary disease and liver metastases is expected to have a significant overlap with some patients receiving multiple episodes of chemotherapy and constitutes a potential confounding factor. Additionally, the type of chemotherapy was not controlled in the study at hand to assess the effect of individual treatment regimes. There was a significantly higher proportion of patients with synchronous disease and multiple liver metastases that explains a particularly higher risk of disease recurrence. However, the high recurrence rates in this subset convey an important message and will potentially help in the selection of patients for high-risk procedures. This multicentre study with varying patient volumes also reflects actual clinical practice and this is the main strength of this study. A future prospective database including molecular profiling and further validation of the proposed nomogram for this particular cohort of BiCRLM is recommended as liver surgeons and oncologists will be continuously challenged with the selection of patients for newer surgical options such as partial liver transplantation (RAPID) and liver transplantation procedures.

Despite the high recurrence rates following liver resection, OS rates following resection of BiCRLM are encouraging. The BiCRLM-OS nomogram can be used in the selection of patients for higher risk surgical options as well as preoperative and postoperative counselling.

## BiCRLM study collaborators

Alexander Novotny (Technical University, Munich, Germany); Alfred Kow (National University of Singapore, Singapore); Amar Kourdouli (University Hospitals of Leicester, Leicester, UK); Andrea Belli (l'Istituto Nazionale Tumori di Napoli, Napoli, Italy); Andres Valdivieso (University of Basque Country, Bilbao, Spain); Angus Hann (Queen Elizabeth Hospital, Birmingham, UK); Ángela de la Hoz Rodríguez (Hospital Universitario La Princesa, Madrid, Spain); Anisa Nutu Oona (Queen Elizabeth Hospital, Birmingham, UK); Andreas Pascher (University Hospital Würzburg,Wurzburg, Germany); Antonio Frena (Central Hospital of Bolzano, Bolzano, Italy); Arpad Ivanecz (University Medical Center Maribor, Slovenia); Asmus Heumann (University Hospital Hamburg-Eppendorf, Germany); Ayaya Alonso Alvarado (Hospital Universitario Nuestra Senora de Candelaria,Santa Cruz de Tenerife, Spain); Ayrat Kaldarov (Vishnevsky Centre of Surgery, Moscow, Russia); Bart Bracke (Antwerp University Hospital, Belgium); Bart Hendrikx (Ghent University Hospital, Ghent, Belgium); Benjamin Struecker (Universitätsklinikum Münster, Germany); Bergthor Bjornsson (Linkoping University, Linkoping, Sweden); Carmen Cutolo (University of Salerno, Ficsiano, Italy); Carlo Frola (Royal Free Hospital, London, UK); Carmen Payá-Llorente (Hospital Universitario Doctor Peset, Valencia, Spain); Carlos Domingo-del Pozo (Hospital Universitario Doctor Peset, Valencia, Spain); Catherine Teh (Makati Medical Center, Phillipines); Christian Stöss (Technical University, Munich, Germany); Claudio Ricci (Azienda Ospedaliero-Universitaria di Bologna, Bologna, Italy); Cornelis Verhoef (Erasmus MC Cancer Institute, Rotterdam, Netherlands); Cristina Dopazo (Hospital Universitario Vall d'Hebron, Barcelona, Spain); Daniel Galun (University Clinical Centre of Serbia, Belgrade); Daniel Hartmann (Technical University, Munich, Germany); David Martin (Lausanne University Hospital, Laussane, Switzerland); Diego Greatti Vaz da Silva (AC Camargo Cancer Center, Brazil); Dimitri Dorcaratto (Hospital Clínico, University of Valencia, Biomedical Research Institute (INCLIVA), Valencia, Spain); Dimitrios Magouliotis (University of Thessaly, Greece); Dimitrios Moris (Laikon General Hospital, Athens, Greece); Dimitrios Symeonidis (University Hospital of Larissa, Larissa, Greece); Dimitrios Zacharoulis (University Hospital of Larissa, Larissa, Greece); Dursun Bugra (Koc University Hospital, Istanbul, Turkey); Dolores Lopez-Garnica (Hospital Universitario Vall d'Hebron, Barcelona, Spain); Eduard Jonas (University of Cape Town Health Sciences Faculty and Groote Schuur Hospital, Cape Town, South Africa); Edoardo Maria Muttillo (Nouvel Hospital Civil, Strasbourg, France); Edoardo Saladino (UOC di Chirurgia Generale Oncologica – AO Papardo – Messina); Elsa Francisco (Hospital Fernando Fonseca, Amadora, Portugal); Ela Hutten (Amphia Hospital, The Netherlands); Emilio De Raffele (Azienda Ospedaliero-Universitaria di Bologna, Bologna, Italy); Emanuele Felli (Service Chirurgie Digestive et Transplantation Hepatique Hospital, Trousseau, France); Emre Balik (Koc University Hospital, Istanbul, Turkey); Emre Bozkurt (Koc University Hospital, Istanbul, Turkey); Evangelos Felekouras (Laikon General Hospital, Athens, Greece); Erman Sobutay (Koç Foundation American Hospital, Istanbul, Turkey); Ernesto Sparrelid (Karolinska University Hospital, Stockholm, Sweden); Fabrizio Romano (San Gerado Hospital, Monza, Italy); Felipe José Fernández Coimbra (AC Camargo Cancer Center, Brazil); Fiorentini Guido (San Raffaele Hospital, Milan, Italy); Florian Primavesi (Medical University Innsbruck, Austria); Francesco Izzo (l'Istituto Nazionale Tumori di Napoli, Napoli, Italy); Frederik Berrevoet (Ghent University Hospital, Ghent, Belgium); Gaetano Piccolo (San Paolo Hospital, Milan, Italy); Gaëtan-Romain Joliat (Lausanne University Hospital, Laussane, Switzerland); Gary Middleton (Queen Elizabeth Hospital, Birmingham, UK); Georgios Makridis (St Josefs-Hospital, Wiesbaden, Germany); Georgios C. Sotiropoulos (Evaggeslismos General Hospital, Athens, Greece); Giuseppe Garcea (University Hospitals of Leicester, Leicester, UK); Glen Booney (National University of Singapore, Singapore); Ho-Seong Han (Seoul National University Bundang Hospital, Bundang, South Korea); Ibrahim Halil Ozata (Koç University, Istanbul, Turkey); Jai Young Cho (Seoul National University Bundang Hospital, Bundang, South Korea); Jiri Pudil (Military University Hospital Prague, Prague, Czech Republic); John Hammond (Freeman Hospital, NewCastle, UK); Jorge Brian Torres (Hospital Universitario de Canarias, Tenerife, Spain); Jun Li (University Hospital Hamburg-Eppendorf, Germany); Joerg-Matthias Pollok (Royal Free Hospital, London, UK); Khaled Ammar (Freeman Hospital, NewCastle, UK); Kostiantun Kopchak (National cancer institute, Ukraine); Kojiro Taura (Kyoto University Graduate School of Medicine, Kyoto, Japan); Kursat Serin (Istanbul University, Istanbul, Turkey); Krishna Menon (King's College Hospital, London, UK); Krzysztof Zieniewicz (Medical University of Warsaw, Poland); Leticia Perez-Santiago (Hospital Clínico, University of Valencia, Biomedical Research Institute (INCLIVA), Valencia, Spain); Linda Lundgren (County Council of Östergötland, Linköping, Sweden); Lissa Wullaert (Amphia Hospital, Department of Surgery, Netherlands); Luca Alderghetti (San Raffaele Hospital, Milan, Italy); Luis Abreu De Carvalho (Ghent University Hospital, Ghent, Belgium); Madita-Magdalena Tschöegl (Clinic Favoriten, Vienna, Austria); Marco Marino (Azienda Ospedaliera Ospedali Riuniti Villa Sofia-Cervello, Palermo, Italy); María Aránzazu (Hospital Universitario Nuestra Senora de Candelaria,Santa Cruz de Tenerife, Spain); Markus Ammann (County Hospital Wiener Neustadt, Vienna. Austria); Aranzazu Varona-Bosque (ICMDM, Hospital Clinic, Barcelona, Spain); Mario Giuffrida (Parma University Hospital, Parma, Italy); Mattia Garancini (San Gerado Hospital, Monza, Italy); Mauro Alessandro Scotti (San Gerado Hospital, Monza, Italy); Matteo Barabino (San Paolo Hospital, Milan, Italy); Marc Bernon (University of Cape Town Health Sciences Faculty and Groote Schuur Hospital, Cape Town, South Africa); Matteo Cescon (S.Orsola-Malpighi Hospital, Bologna, Italy); Marcello Di Martino (Hospital Universitario La Princesa, Madrid, Spain); Marcello Maestri (IRCCS Policlinico San Matteo Foundation, Italy); Marco Massani (Teviso Regional Hospital, Treviso, Italy); Maria Sotiropoulou (Evangelismos General Hospital, Athens, Greece); Maria Teresa Abadia Forcen (Hospital Universitario Miguel Servet, Zaragoza, Spain); Maria-Carmen Fernandez-Moreno (Hospital Clínico, University of Valencia, Biomedical Research Institute (INCLIVA), Valencia, Spain); Mario Serradilla-Martín (Hospital Universitario Miguel Servet, Zaragoza, Spain); Marko Zivanovic (University Clinical Centre of Serbia, Belgrade); Marta Gutiérrez-Díez (Hospital Universitario Miguel Servet, Zaragoza, Spain); Melek Buyuk (Istanbul University, Istanbul, Turkey); Michail Vailas (Laikon General Hospital, Athens, Greece); Mitesh Sharma (Royal Free Hospital, London, UK); Mizelle D'Silva (Jaslok Hospital, Mumbai, India); Mladjan Protic (University of Novi Sad, Novi Sad, Serbia); Mohammad Hossein Fard-Aghaie (University Hospital Hamburg-Eppendorf, Germany); Lissa Wullaert (Amphia Hospital, Department of Surgery, Netherlands); Nagappan Kumar (University Hospital of Wales, Cardiff, UK); Narimã Marques (AC Camargo Cancer Center, Brazil); Nefeli Tomara (National and Kapodistrian University of Athens. Athens, Greece); Nicholas G Mowbray (University Hospital of Wales, Cardiff, UK); Nicolas Demartines (Lausanne University Hospital, Laussane, Switzerland); Nikolaos Machairas (National and Kapodistrian University of Athens. Athens, Greece); Offir Ben-Ishay (Rambam Health Care Campus, Israel); Oleksandr Kvasivka (National cancer institute, Ukraine); Olivera Krsmanovic (University of Novi Sad, Novi Sad, Serbia); Orhan Bilge (American Hospital, Istanbul, Turkey); Pablo Sancho-Pardo (Hospital Universitario Miguel Servet, Zaragoza, Spain); Pal-Dag Line (Oslo University Hospital, Oslo); Pascale Tinguely (Royal Free Hospital, London, UK); Patrick Pessaux (University Hospital of Strasbourg, France); Per Sandstrom (Linkoping University, Linkoping, Sweden); Peter Lodge (St James's University Hospital, Leeds, UK); Raffaele Dalla Valle (Parma University Hospital, Parma, Italy); Roger Homs (Hospital de la Santa Creu i Sant Pau, Barcelona, Spain); Robert Sutcliffe (Queen Elizabeth Hospital, Birmingham, UK); Sanja Lob (University Hospital Würzburg,Wurzburg, Germany); Santiago Sánchez-Cabús (Hospital de la Santa Creu i Sant Pau, Barcelona, Spain); Shadi Katou (University Hospital Würzburg,Wurzburg, Germany); Shinya Okumura (Kyoto University Graduate School of Medicine, Kyoto, Japan); Etsuro Hatano (Kyoto University Graduate School of Medicine, Kyoto, Japan); Spela Turk (University Medical Center Maribor, Slovenia); Stefan Farkas (St Josefs-Hospital, Wiesbaden, Germany); Stefan Patauner (Central Hospital of Bolzano, Bolzano, Italy); Stefan Stättner (Salzkammergut Klinikum OÖG Vöcklabruck, Innsbruck, Austria); Stefan Löb (University Hospital Würzburg,Wurzburg, Germany); Stephanie Truant (Chru de Lille, France); Stylianos Kapiris (Evaggeslismos General Hospital, Athens, Greece); Tom Gallagher (St Vincent's University Hospital, Dublin, Ireland); Tereza Kocisova (Military University Hospital Prague, Prague, Czech Republic); Thomas Gruenberger (Clinic Favoriten, Vienna, Austria); Tommaso Stecca (Teviso Regional Hospital, Treviso, Italy); Thiery Chapelle (Antwerp University Hospital, Belgium); Teresa Abadía-Forcén (Hospital Universitario Miguel Servet, Zaragoza, Spain); Víctor Molina (Hospital de la Santa Creu i Sant Pau, Barcelona, Spain); Valeriia Sumarokova (National cancer institute, Ukraine); Yannick Meyer (Erasmus MC Cancer Institute, Rotterdam, Netherlands).

## Supplementary Material

zrad085_Supplementary_Data

## Data Availability

Data will be available from the corresponding author, if the request is approved by the study scientific committee.
