# Corrigendum to: Development and validation of a novel risk score to predict overall survival following surgical clearance of bilobar colorectal liver metastases

**DOI:** 10.1093/bjsopen/zrad164

**Published:** 2023-12-27

This is a correction to: Bobby V M Dasari, Dimitri Raptis, Nicholas Syn, Alejandro Serrablo, Jose Manuel Ramia, Andrea Laurenzi, Christian Sturesson, Timothy M Pawlik, Ajith K Siriwardena, Mickael Lesurtel, on behalf of the Scientific Committee of E-AHPBA, Development and validation of a novel risk score to predict overall survival following surgical clearance of bilobar colorectal liver metastases, *BJS Open*, Volume 7, Issue 5, October 2023, zrad085, https://doi.org/10.1093/bjsopen/zrad085

In the originally published version of this manuscript the group name **BiCRLM study collaborators** was erroneously missed from the author byeline. This has now been emended within the article.

