# Supplementary material for: Development and validation of a novel risk score to predict overall survival following surgical clearance of bilobar colorectal liver metastases
Source: BJS Open. 2023 Sep 21;7(5):zrad085. doi: 10.1093/bjsopen/zrad085 (PMC10516618; doi:10.1093/bjsopen/zrad085)
Supplement: zrad085_Supplementary_Data [file zrad085_supplementary_data.docx]

**Title**

**Development and validation of a novel risk score to predict overall survival following surgical clearance of bilobar colorectal liver metastases**

Bobby VM Dasari FRCS^1,2^, Dimitri Raptis FRCS^3^, Nicholas Syn MBBS^4^, Alessandro Serrablo FACS^5^, Jose Ramia-Angel FRCS^6^, Andrea Laurenzi FRCS^7^, Christian Sturesson MD^8^, Tim Pawlik FACS^9^, Ajith K Siriwardena FRCS^10^, Mickael Lesurtel MD^11^, on behalf of the Scientific Committee of EAHPBA

Affiliations:

1. University of Birmingham, Institute of Immunology and Immunotherapy, University of Birmingham

2. Department of HPB Surgery and Liver Transplantation, Queen Elizabeth Hospital, Birmingham, UK

3. Department of HPB Surgery and Liver Transplantation, Royal Free Hospital, London, UK

4. Department of HPB Surgery and Liver Transplantation, National University of Singapore, Singapore

5. HBP Surgical Division, Miguel Servet University Hospital, Zaragoza, Spain

6. Department of Hepatobiliary Surgery and Liver Transplantation, Hospital General Universitario de Alicante, Alicante, Spain

7. Hepatobiliary Surgery and Organ Transplantation, IRCCS Azienda Ospedaliero-Universitaria di Bologna, Bologna, Italy

8. Department of Hepatobiliary and Liver Transplantation, Karolinska Institute, Karolinska, Sweden

9. Division of Surgery, Oncology, and Health Services Management and Policy, The Ohio State University Wexner Medical Center

10. Department of Hepatobiliary surgery, Manchester Royal Infirmary, Manchester, UK

11. Department of HPB Surgery & Liver Transplantation, Beaujon Hospital - University of Paris Cité, France

***Corresponding author:***

Bobby VM Dasari MS, MScEd, FRCS, University of Birmingham, Institute of Immunology and Immunotherapy, University of Birmingham; Department of HBP and Liver Transplantation Surgery, Queen Elizabeth Hospital, Edgbaston, Birmingham, UK

[Bobby.dasari@uhb.nhs.uk](mailto:Bobby.dasari@uhb.nhs.uk); [Bobby.dasari@yahoo.com](mailto:Bobby.dasari@yahoo.com); [b.dasari@bham.ac.uk](mailto:b.dasari@bham.ac.uk)

#DasariVm

**Supplementary Materials - Index**

| **Supplementary Form 1**  **Case Report Form Page 3** |  |
| --- | --- |
| **Supplementary Form 2**  **TRIPOD checklist Page 9** |  |
| **Supplementary Table 1**  **List of participating centers Page 10**  **Supplementary Table 2. Page 12**  **Predicted OS based on the score from nomogram and cured proportion from AFT model** |  |

**Supplementary Form 1: Case Report Form**

Case ID

Please add the unique case number as an identifier. Do not use the hospital number or patient identifiers. Please keep a separate list of anonymized case numbers linking to the patient hospital number somewhere safe at your institution. This will help you identify patients in the CRF for further editing in the future if needed.

**Age at** the time of liver resection ______ years

**Gender** M / F

Height _____ cm, Weight _____ Kg

BMI. ______ kg/m2

**ASA score:**

ASA 1: Able to carry out all normal activity without restriction

ASA 2: Restricted in strenuous activity but ambulatory and able to carry out light work

ASA 3: Symptomatic and in a chair or in bed for greater than 50% of the day but not bedridden

ASA 4: Completely disabled, cannot carry out any self-care, totally confined to bed or chair

**WHO Performance status**: 0 1 2 3 4

**Primary tumor characteristics:**

Site of Primary: Right Left. Transverse. Rectum

T-stage of primary (histological):

Tis. T1 T2 T3 T4 Unknown

T stage of primary (radiological):

Tis. T1 T2 T3 T4 Unknown

N-stage of primary (histological):

N0 N1 N2 Unknown

N-stage of primary (radiological)

N0 N1 N2 Unknown

Resection margins (if resected)

R0 R1 R2

RAS mutation of primary

KRAS BRAF NRAS PIK3CA KRAS wild type Unknown

Days from diagnosis of colorectal cancer until resection of the primary * days

Online date duration calculator: https://[www.timeanddate.com/date/duration.html](http://www.timeanddate.com/date/duration.html)

Chemotherapy given prior to primary resection?

Yes No Unknown

If chemotherapy given after primary resection, please indicate type and cycles: ________

Status of primary at the time of resection:

Insitu

Resected

Completely responded

**Liver metastases characteristics:**

Type of liver disease at the time of diagnosis

Resectable

Borderline resectable

Unresectable

Synchronicity of liver metastases

Synchronous Metachronous

Days from diagnosis of primary until diagnosis of liver metastases * days

Extrahepatic disease at the time of diagnosis of liver metastases (multiple options)

No extra hepatic disease

Lung

Peritoneum

Unresected primary

Recurrence at resection of primary

If yes, was the extrahepatic disease considered completely treated prior to surgery for liver metastases (multiple options)

Resection

Ablation

SABR (stereotactic ablative radiotherapy)
Chemotherapy

No treatment given

# Imaging prior to liver resection

Total number of liver metastases on imaging prior to resection ____

Number of lesions on the right lobe (Seg 5,6,7,8) ______

Number of lesions in S4: ____

Number in left lateral segments (S2,S3): _____

Size of the largest lesion : _____mm

Radiological response to chemotherapy:

All lesions responded

Some responded and some increased

Some responded and there were new lesions

Some disappeared

Remained the same

All disappeared

Not applicable

RECIST criteria:

For more information, please see: https://en.wikipedia.org/wiki/Response_evaluation_criteria_in_solid_tumors

Unknown

Complete response

Partial response

Stable disease

Progressive disease

Chemotherapy given prior to resection of liver metastases. Yes. No

If Chemotherapy given prior to liver resection, type, number of cycles and duration of chemo:

**Details of liver surgery:**

**Type of liver resection:**

Multiple wedge resections

Right hepatectomy

Left hepatectomy

Right extended

Left extended

Left lateral sectionectomy

Posterior liver resection

Two stage hepatectomy with PVE

Two stage resection with PVL

Two stage resection with ALPPS

Other: __________

(Two stage hepatectomy indicates two operations with liver resection e.g. Stage 1 resection followed by PVE and then followed by stage 2 liver resection or ALPPS: Associating liver partition and portal vein ligation for staged hepatectomy)

If Wedge resections were performed, indicate the total number and segments: _____________

If intraoperative ablations were performed, indicate the total number and segments: ___________

Were there any disappearing liver lesions that were left in situ: _________

Were there any known liver lesions not identified at the time of liver resection: ________

**PVE strategies:**

PVE performed: **Yes No**

Sequence of PVE:

No PVE performed

PVE followed by liver resection

PVE and HVE followed by liver resection

Stage 1 liver resection, followed by PVE, followed by stage 2 liver resection

Stage 1 liver resection, followed by PVE and HVE, followed by stage 2 liver resection

(PVE: portal vein embolization; HVE: Hepatic vein embolization)

Did the patient proceed to second stage resection? Yes No

If two stage procedure, days from stage 1 to stage 2________ days

Were there any new lesions identified or disease progression between stage 1 and stage 2? ______

# Histopathology

# Margin status of the single stage or two stage resection (please record the highest margin status. If lesion 1= R0, Lesion 2=R1, Lesion 3=R2; please record margin status as R2)

R0 (>/= 1mm distance of tumour from resection margin)

R1 (<1mm from resection margin)

R2 (macroscopically positive margin)

Mutant RAS of the metastases

Unknown KRAS BRAF NRAS PIK3CA

Evidence of response to chemotherapy on histology:

Yes No Unknown

If yes, grade of response: _________

# Early postoperative course

Clavien-Dindo Classification of postoperative complications until 90 days postoperatively.

*This refers to liver resections only, not postoperative complications following surgery for the primary. If two stage hepatectomies performed, please indicate the cumulative complications until 90 days from the second stage liver resection. For example, if a patient developed atrial fibrillation requiring medical cardioversion postoperatively after stage 1 and developed chest infection requiring antibiotics after stage 2, then indicate x2 grade 2 complications. If a patient was readmitted to any hospital with an additional complication within 90 days postoperatively, please include the grade of complication as well. For more information, see: https://*[*www.assessurgery.com/clavien-dindo-classification/*](http://www.assessurgery.com/clavien-dindo-classification/)

Grade 1 Yes No If yes, number of complications: _____

Grade 2 Yes No. If yes, number of complications: _____

Grade 3a Yes No If yes, number of complications: _____

Grade 3b Yes No If yes, number of complications: _____

Grade 4a Yes No If yes, number of complications: _____

Grade 4b Yes No If yes, number of complications: _____

Grade 5 - death Yes No

Length of post operative hospital stay in days: __________________

For two stage hepatectomies, please indicate the cumulative hosospital stay. If a patient was readmitted to the hospital within 90 days postoperatively, then also include the cumulative hospital stay for both the primary operation and readmission.

Chemotherapy offered after liver resection: Yes No

If chemotherapy offered after liver resection, please indicate type and number of cycles: _______

**Follow up**

Patient status:

Alive without disease

Alive with liver only disease

Alive with extrahepatic disease only

Alive with liver and extrahepatic disease

Dead from cancer

Dead due to another cause

Dead from unknown cause

Patient status unknown

The patient status indicates whether the patient was last seen alive or dead at the hospital, followed up at the outpatient clinic, family doctor, or confirmed after being contacted by phone. Below you may indicate the number of days from operation until last follow up or death. If the patient underwent a two stage hepatectomy, please indicate the number of days from stage 1 liver resection until death or end of follow up.

Days from operation to last follow up or death ________ days

i.e. days from operation to death or last follow up record. This value (number of days) may indicate the time form operation to the last follow up record for alive patients or the time from operation to death for those that died.

Date duration calculator

**Is there disease recurrence?** Yes. No

If yes, indicate the site of first recurrence (multiple options):

In the liver at site of previously treated (resection/ablation) lesion

In the liver but not at the site of previously treated lesion

In the lungs

Peritoneal

At primary resection site

Elsewhere

Days from operation until any site disease recurrence in days: ________

If no recurrence, please leave blank. If two staged hepatectomy, days from stage 1 until recurrence

Days from operation until Liver recurrence in days: _______

If no recurrence, please leave blank. If liver was the first site of recurrence, indicate the same value here as above. If two staged hepatectomy, days from stage 1 until recurrence.

Treatment of recurrent liver disease (multiple options)

Further surgery

Ablation

Stereotactic ablative radiotherapy (SABR)

Selective Internal radiation therapy (SIRT)

Chemotherapy

Other

Unknown

Treatment of extrahepatic disease (multiple options)

Ablation

Surgery

Chemotherapy

Other

Unknown

Additional comments: _____

**Supplementary Form 2: TRIPOD Checklist:**

| **Section/Topic** | **Item** | **Checklist Item** | **Page** |
| --- | --- | --- | --- |
| **Title and abstract** | | | |
| Title | 1 | Identify the study as developing and/or validating a multivariable prediction model, the target population, and the outcome to be predicted. | Page 1 |
| Abstract | 2 | Provide a summary of objectives, study design, setting, participants, sample size, predictors, outcome, statistical analysis, results, and conclusions. | Page 3 |
| **Introduction** | | | |
| Background and objectives | 3a | Explain the medical context (including whether diagnostic or prognostic) and rationale for developing or validating the multivariable prediction model, including references to existing models. | Page 4 |
|  | 3b | Specify the objectives, including whether the study describes the development or validation of the model or both. | Page 4, 5 |
| **Methods** | | | |
| Source of data | 4a | Describe the study design or source of data (e.g., randomized trial, cohort, or registry data), separately for the development and validation data sets, if applicable. | Page 5 |
|  | 4b | Specify the key study dates, including start of accrual; end of accrual; and, if applicable, end of follow-up. | Page 5 |
| Participants | 5a | Specify key elements of the study setting (e.g., primary care, secondary care, general population) including number and location of centres. | Page 5 |
|  | 5b | Describe eligibility criteria for participants. | Page 5 |
|  | 5c | Give details of treatments received, if relevant. | Page 5 |
| Outcome | 6a | Clearly define the outcome that is predicted by the prediction model, including how and when assessed. | Page 5, 6 |
|  | 6b | Report any actions to blind assessment of the outcome to be predicted. | NA |
| Predictors | 7a | Clearly define all predictors used in developing or validating the multivariable prediction model, including how and when they were measured. | Page 6 |
|  | 7b | Report any actions to blind assessment of predictors for the outcome and other predictors. | NA |
| Sample size | 8 | Explain how the study size was arrived at. | Page 7 |
| Missing data | 9 | Describe how missing data were handled (e.g., complete-case analysis, single imputation, multiple imputation) with details of any imputation method. | NA |
| Statistical analysis methods | 10a | Describe how predictors were handled in the analyses. | Page 6 |
|  | 10b | Specify type of model, all model-building procedures (including any predictor selection), and method for internal validation. | Page 6 |
|  | 10d | Specify all measures used to assess model performance and, if relevant, to compare multiple models. | Page 6 |
| Risk groups | 11 | Provide details on how risk groups were created, if done. | Page 6 |
| **Results** | | | |
| Participants | 13a | Describe the flow of participants through the study, including the number of participants with and without the outcome and, if applicable, a summary of the follow-up time. A diagram may be helpful. | Page 7 |
|  | 13b | Describe the characteristics of the participants (basic demographics, clinical features, available predictors), including the number of participants with missing data for predictors and outcome. | Page 7 |
| Model development | 14a | Specify the number of participants and outcome events in each analysis. | Page 7 |
|  | 14b | If done, report the unadjusted association between each candidate predictor and outcome. | Page 8,9  Table 3 |
| Model specification | 15a | Present the full prediction model to allow predictions for individuals (i.e., all regression coefficients, and model intercept or baseline survival at a given time point). | Table 3,4  Figures 3,4,5 |
|  | 15b | Explain how to the use the prediction model. | Page 9 |
| Model performance | 16 | Report performance measures (with CIs) for the prediction model. | Page 9 and Table 4,5 |
| **Discussion** | | | |
| Limitations | 18 | Discuss any limitations of the study (such as nonrepresentative sample, few events per predictor, missing data). | Page 11 |
| Interpretation | 19b | Give an overall interpretation of the results, considering objectives, limitations, and results from similar studies, and other relevant evidence. | 11,12 |
| Implications | 20 | Discuss the potential clinical use of the model and implications for future research. | 11,12 |
| **Other information** | | | |
| Supplementary information | 21 | Provide information about the availability of supplementary resources, such as study protocol, Web calculator, and data sets. | Page 5,9 |
| Funding | 22 | Give the source of funding and the role of the funders for the present study. | Page 1 |

**Supplementary Table 1: List of participating units / centers**

| Participating Unit | Numbers contributed |
| --- | --- |
| AC Camargo Cancer Center, Brazil | 39 |
| Amphia Hospital, Department of Surgery, Netherlands | 10 |
| Antwerp University Hospital, Belgium | 42 |
| Azienda Ospedaliero-Universitaria di Bologna, Bologna, Italy | 11 |
| Chru de Lille, France | 43 |
| County Hospital Wiener Neustadt, Vienna. Austria | 5 |
| Erasmus MC Cancer Institute, Rotterdam, Netherlands | 97 |
| Freeman Hospital, NewCastle, UK | 5 |
| Ghent University Hospital, Belgium | 14 |
| Hospital Clı´nico, University of Valencia, Valencia, Spain | 19 |
| Hospital de la Santa Creu i Sant Pau, Barcelona, Spain | 9 |
| Hospital Fernando Fonseca, Portugal | 6 |
| Hospital Universitario de Canarias, Spain | 12 |
| Hospital Universitario Doctor Peset, Valencia, Spain | 8 |
| Hospital Universitario La Princesa, Madrid, Spain | 12 |
| Hospital Universitario Miguel Servet, Zaragoza, Spain | 5 |
| Hospital Universitario Miguel Servet, Zaragoza, Spain | 19 |
| Hospital Universitario Miguel Servet, Zaragoza, Spain | 6 |
| Hospital Universitario Vall d'Hebron, Barcelona, Spain | 30 |
| HPB center, Health Network, Vienna, Austria | 24 |
| HPB Referral Center, Treviso, Italy | 8 |
| IRCCS Policlinico San Matteo Foundation, Italy | 3 |
| Istanbul Faculty of Medicine, Istanbul, Turkey | 9 |
| Koc University, Istanbul, Turkey | 37 |
| Kyoto University, Kyoto, Japan | 18 |
| l’Istituto Nazionale Tumori di Napoli, Napoli, Italy | 17 |
| Laikon General Hospital, Athens, Greece | 2 |
| Lausanne University Hospital CHUV, Lausanne, Switzerland | 20 |
| Linkoping University, Linkoping, Sweden | 26 |
| Linkoping University, Linkoping, Sweden | 24 |
| Linköping University, Linkoping, Sweden | 31 |
| Makati Medical Center, Philippines | 1 |
| Medical University Innsbruck, Innsbruck, Austria | 7 |
| Medical University of Warsaw, Poland | 17 |
| Military University Hospital Prague, Prague, Czech Republic | 7 |
| National and Kapodistrian University of Athens, Athens, Greece | 11 |
| National cancer institute, Kyiv, Ukraine | 9 |
| Nouvel Hospital Civil, Strasbourg, France | 3 |
| Parma University Hospital, Parma, Italy | 3 |
| Queen Elizabeth Hospital, Birmingham, UK | 18 |
| Queen Elizabeth Hospital, Birmingham, UK | 23 |
| Rambam Health Care Campus, Israel | 3 |
| Royal Free Hospital, London, UK | 65 |
| S.Orsola-Malpighi Hospital, Bologna, Italy | 71 |
| San Gerado Hospital, Monza, Italy | 11 |
| San Paolo Hospital, Milan, Italy | 18 |
| San Raffaele Hospital, Milan, Italy | 26 |
| Santa Cruz de Tenerife, Spain | 13 |
| Seoul National University Bundang Hospital, Bundang, South Korea | 27 |
| Service Chirurgie Digestive et Transplantation Hepatique Hospital, Trousseau, France | 6 |
| St James University Hospital, Leeds, UK | 38 |
| St Josefs-Hospital, Wiesbaden, Germany | 9 |
| St Vincent’s University Hospital, Dublin, Ireland | 6 |
| Technical University, Munich, Germany | 5 |
| Universitätsklinikum Münster, Germany | 2 |
| University Clinic for Digestive Surgery, Belgrade, Serbia | 11 |
| University Hospital Hamburg-Eppendorf, Hamburg, Germany | 20 |
| University Hospital of Wales, Cardiff, UK | 20 |
| University Hospital Würzburg, Wurzburg, Germany | 33 |
| University Hospital, Stockholm, Sweden | 2 |
| University Hospitals of Leicester NHS Trust, Leicester, UK | 7 |
| University Medical Centre Maribor, Slovenia | 66 |
| University of Basque Country, Bilbao, Spain | 32 |
| University of Cape Town and Groote Schuur Hospital, Cape Town, South Africa | 10 |
| University of Milan, Milan, Italy | 11 |
| University of Novi Sad, Novi Sad, Serbia | 20 |
| University of Thessaly, Thessaly, Greece | 4 |
| UOC di Chirurgia Generale Oncologica, AO Papardo, Messina, Italy | 11 |

**Supplementary Table 2. Predicted OS based on the score from nomogram and cured proportion from AFT model**

| Total score | Median OS (months) | 1-yr OS | 2-yr OS | 3-yr OS | 5-yr OS | 10-yr OS | Cured proportion |
| --- | --- | --- | --- | --- | --- | --- | --- |
| 1 | NR | 99.9% | 99.7% | 99.1% | 97.2% | 90.2% | 74.2.% |
| 2 | NR | 99.9% | 99.4% | 98.5% | 95.5% | 86.0% | 68.5% |
| 3 | NR | 99.8% | 99.0% | 97.4% | 93.1% | 80.7% | 60.8% |
| 4 | NR | 99.7% | 98.3% | 95.9% | 89.8% | 74.3% | 52.6% |
| 5 | 193.5 | 99.4% | 97.1% | 93.7% | 85.5% | 66.9% | 44.0% |
| 6 | 150.2 | 99.0% | 95.4% | 90.5% | 80.0% | 58.7% | 35.6% |
| 7 | 120.2 | 98.2% | 92.8% | 86.3% | 73.5% | 50.2% | 27.6% |
| 8 | 96.0 | 97.0% | 89.4% | 81.0% | 66.0% | 41.7% | 20.4% |
| 9 | 73.6 | 95.3% | 85.0% | 74.7% | 57.7% | 33.6% | 14.2% |
| 10 | 61.1 | 92.8% | 79.4% | 67.4% | 49.3% | 26.1% | 10.0% |
| 11 | 47.8 | 89.3% | 72.8% | 59.3% | 40.8% | 19.6% | 6.0% |
| 12 | 37.1 | 84.7% | 65.2% | 50.8% | 32.7% | 14.2% | 3.1% |
| 13 | 28.4 | 79.1% | 57.0% | 42.2% | 25.3% | 10.0% | 2.2% |
| 14 | 23.9 | 72.4% | 48.4% | 34.2.% | 18.9% | 6.7% | NE |
| 15 | 18.7 | 65.6% | 45.5% | 26.1% | 13.7% | 3.5% | NE |
| 16 | 14.8 | 57.4% | 31.2% | 19.7% | 10.0% | 2.5% | NE |
| 17 | 11.9 | 48.9% | 23.9% | 14.3% | 6.4% | 1.8% | NE |
| 18 | 9.2 | 40.4% | 17.7% | 10.0% | 4.1% | 0.5% | NE |
| 19 | 7.4 | 32.3% | 12.7% | 6.7% | 2.5% | 0.0% | NE |
| 20 | 5.8 | 25.0% | 8.8% | 4.3% | 1.5% | 0.0% | NE |
| 21 | 4.6 | 18.7% | 2.7% | 2.7% | 0.1% | 0.0% | NE |
| 22 | 3.8 | 13.5% | 3.7% | 1.6% | 0.0% | 0.0% | NE |
| 23 | 2.9 | 9.3% | 2.3% | 1.0% | 0.0% | 0.0% | NE |
| 24 | 2.3 | 6.2% | 1.3% | 0.5% | 0.0% | 0.0% | NE |
| 25 | 2.0 | 4.0% | 0.7% | 0.0% | 0.0% | 0.0% | NE |
| 26 | 1.5 | 2.5% | 0.4% | 0.0% | 0.0% | 0.0% | NE |
| 27 | 0.9 | 1.5% | 0.2% | 0.0% | 0.0% | 0.0% | NE |
| 28 | 0.4 | 0.8% | 0.0% | 0.0% | 0.0% | 0.0% | NE |
